# Classification, Mechanisms of Action, and Therapeutic Applications of Inhibitory Oligonucleotides for Toll-Like Receptors (TLR) 7 and 9

**DOI:** 10.1155/2010/986596

**Published:** 2010-05-18

**Authors:** Petar S. Lenert

**Affiliations:** Department of Internal Medicine, Division of Rheumatology, Carver College of Medicine, The University of Iowa, 200 Hawkins Drive, Iowa City, Iowa 52242, USA

## Abstract

Our immune defense depends on two specialized armed forces. The innate force acts as an alarm mechanism that senses changes in the microenvironment through the recognition of common microbial patterns by Toll-like receptors (TLR) and NOD proteins. It rapidly generates an inflammatory response aimed at neutralizing the intruder at the mucosal checkpoint. The innate arm also communicates this message with more specialized adaptive forces represented by pathogen-specific B cells and T cells. Interestingly, B cells also express some innate sensors, like TLR7 and TLR9, and may respond to bacterial hypomethylated CpG motifs and single-stranded RNA viruses. Intracellular nucleic acid sensing TLRs play an important role in the pathogenesis of Systemic Lupus Erythematosus (SLE). In this review, we describe recent achievements in the development of oligonucleotide—(ODN)-based inhibitors of TLR9 and/or TLR7 signaling. We categorize these novel therapeutics into Classes G, R, and B based on their cellular and molecular targets. Several short ODNs have already shown promise as pathway-specific therapeutics for animal lupus. We envision their future use in human SLE, microbial DNA-dependent sepsis, and in other autoinflammatory diseases.

## 1. Overview

In this review, we present multiple lines of evidence that short oligonucleotides (ODN) containing stretches of 3–5 guanine nucleotides may act as TLR9-specific antagonists. We define their optimal sequence requirements, discuss the importance of secondary structures, present evidence of their efficacy in animal models of lupus and sepsis in vivo, and offer a new classification based on their mechanisms of action and cellular selectivity. We further discuss the ability of phosphorothioate-modified ODNs to act as TLR7 antagonists.

## 2. Toll-Like Receptor 9 as an Immune Sensor of Unmethylated CpG-DNA

Cells of our innate immune system can be activated by bacterial DNA, but not by our own DNA [1]. When unmethylated CpG sequences flanked with two purines at the 5′ end and with two pyrimidines at the 3′ end (so-called CpG motif) were found to be necessary for bacterial DNA-induced immune activation [2–5], the whole field of oligonucleotide research exploded culminating in the discovery of the TLR9 as a receptor responsible for CpG-ODN (and bacterial DNA) action [6, 7]. This effect was recently found to be heavily dependent on DNA sugar backbone recognition by TLR9 [8]. Even though additional DNA recognition molecules and TLR9-independent pathways were recently discovered [9–15], TLR9 itself appears to be both necessary and sufficient for observed immunostimulatory effect of CpG-containing ODNs (reviewed in [3]). Interestingly, TLR9 has relatively limited distribution and in humans is found exclusively in Type I interferon-producing plasmacytoid dendritic cells and in B cells [16]. In mice, macrophages and myeloid dendritic cells also express high levels of TLR9 and respond to CpG-ODN stimulation [17, 18]. 

Toll-like receptors, including TLR9, warn us of the presence of infection, and the ligand-receptor interaction mobilizes cellular resources to promote an early inflammatory response and to initiate robust adaptive immune response. For example, TLR9-activated B cells enter cell cycle and proliferate, upregulate cell-surface molecules involved in antigen presentation/collaboration with cognate T cells (e.g., CD40, MHC Class II and CD86), and secrete multiple chemokines and proinflammatory cytokines (e.g., IL-6 and TNF-*α*) ([19], reviewed in [3]). B cells also secrete polyclonal IgM and IgG3 [2, 20] and with the T cell help can undergo class switching to other Ig isotypes. Once the immediate danger is neutralized, certain TLR(9)-primed B cells and dendritic cells start making regulatory cytokines, such as IL-10 and TGF-*β* ([20, 21] and Lenert et al., unpublished observation) limiting the ongoing inflammation [21]. In dendritic cells, TLR9 (and TLR7) activation induces among others high levels of type I IFN [22], a cytokine heavily implicated in the pathogenesis of Systemic Lupus Erythematosus and Sjögren's syndrome [23–26]. Thus, innate activation through TLRs stands at the cross-roads between innate and adaptive immunity, and if left unchecked may cause chronic immune stimulation and autoimmunity. For example, expansion of transgenic rheumatoid factor-specific B cells in lupus-prone MRL-Fas *^lpr/lpr^* mice is directly dependent on MyD88/TLR expression, but not on T cells [27]. However, the role of TLR9 in the pathogenesis of lupus in this strain of mice remains controversial as some reports suggest that TLR9 may be actually protective rather than pathogenic via induction of regulatory T cells [28, 29]. 

In contrast to the LPS receptor TLR4/MD2, TLR9 is not localized on the cell surface but signals from an interior compartment as first discovered by Wagner's group [30, 31]. In concord with this observation, CpG-ODN- but not LPS-induced intracellular signaling is sensitive to inhibitors of endosomal acidification (e.g., chloroquine) [32]. Cationic peptides such as LL-37 or polymixin may facilitate the uptake of CpG-DNA (including self-DNA) into early endosomes [33]. Once CpG-ODN enters cells, TLR9 undergoes relocation from endoplasmic reticulum to CpG-ODN-containing endosomes [34]. This travel requires a help from the UNC93b1 shuttle protein [35, 36], as mice having a mutation in UNC93b1 fail to respond to intracellular TLR ligands (TLR3, 7 and 9) [37]. After reaching endosomes, TLR9 undergoes its final proteolytic cleavage into a functional receptor [38, 39]. TLR9 exists as a preformed homodimer and CpG-ODN binding promotes its conformational change, bringing the cytoplasmic TIR-like domains close to each other [40]. This allows a recruitment of the key adapter protein MyD88 which initiates a signaling cascade. Following further recruitment of IRAK1/TRAF6 [41, 42], two major signaling pathways are initiated: first through the MAPK/SAPK pathway resulting in AP1 nuclear translocation and second causing NF-*κ*B activation [30, 42, 43], reviewed in [3, 44]. In IFN-*α* producing cells, PI3K, IRF5, and IRF7 are also implicated in CpG-ODN-induced cellular activation [45, 46]. Once these transcription factors bind to their DNA targets, rapid induction of early inflammatory and survival genes follows.

## 3. Discovery of TLR9 Inhibitors

During the course of experiments designed to understand what makes bacterial DNA, but not mammalian DNA, immunostimulatory [1, 4], Pisetsky's group discovered that synthetic oligonucleotides containing poly-G sequences could block bacterial DNA-induced activation [47, 48]. The inhibition was seen at relatively high micromolar concentrations and required that inhibitors were made with the nuclease-resistant phosphorothioate (PS) backbone instead of the natural phosphodiester (PO) backbone. However, these effects were not specific for bacterial DNA-induced activation, as these ODNs could also block other forms of immune stimulation [49]. Others have observed that Poly G-ODNs could suppress tumor cell growth with an IC(50) at 7 micromoles. The inhibition was due to direct binding of Poly-G ODNs to STAT3 preventing its nuclear translocation and interaction with target DNA sequences [50, 51]. As a consequence, the level of survival genes Bcl-2 and Bcl-XL dropped, promoting apoptotic cell death. Thus, poly-G ODNs may represent a new class of chemotherapeutic agents capable of blocking immune activation nonspecifically and promoting apoptotic cell death in tumor cells.

Envisioning application of TLR9 ligands as potential vaccine adjuvants and boosters of antitumor immunity, Krieg's group noticed that certain CpG sequences, like CCGG and methylated CG sequences [52, 53], were not only non-stimulatory, but inhibitory when added to bacterial DNA-stimulated cultures [53]. These inhibitory CpG-sequences were overexpressed in certain strains of adenoviruses (e.g., serotype 2, but not serotype 12) [52]. Therefore, a concept of neutralizing or suppressive CpG-sequences was born [52]. 

Our contribution to the field was to clarify exact sequence requirements for TLR9 inhibition by inhibitory oligonucleotides in mouse and human settings and to study their mechanism of action both in vitro and in vivo. Contribution from other groups will also be mentioned and a modified classification of INH-ODNs will be presented. 

We decoded sequence requirements for inhibitory ODN action in TLR9-activated cells by systematically altering the shortest active 15-mer stimulatory CpG-ODN 2084 (TCCT GACGTT GAAGT) by mutating one or two nucleotides at the time [54, 55]. A CpG to GpC flip created a 100-fold less potent TLR9 agonist compared to the parental molecule; however, the resulting ODN had no TLR9 inhibitory activity by itself. Interestingly, a simple switch from CpG to GpG created an ODN that was capable of blocking both experimental autoimmune encephalomyelitis and spontaneous lupus in mice, as shown by Ho et al. [56, 57]. However, a similar ODN failed to specifically block TLR9-induced stimulation in mouse B cells [55]. Exchanging pyrimidines for guanine nucleotides at the 3′ flank of the CpG motif completely abrogated the stimulatory activity of the prototypic CpG-ODN, creating an inhibitor of the TLR9 signaling. On the other hand, a 5′ flank change from GACGTT to GGCGTT was tolerated quite well in regard to the stimulatory activity. However, when these 3 substitutions were combined in a single ODN, a powerful TLR9 inhibitor-CpG-ODN 2088 (TCCTGGCGGGGAGT) was generated [58, 59]. CpG-ODN-2088 was not only unable to induce TLR9-dependent activation by itself, but could block TLR9-ligand-induced activation at very low nanomolar concentrations. All TLR9-induced biologic outcomes were completely inhibited not only in mouse B cells [59], but also in macrophages and dendritic cells [54]. There was a potency difference of 100–1000-fold between control PS-ODNs and INH-ODNs [59]. At the signaling level, the earliest steps in NF-*κ*B [58] and SAPK/MAPK/AP1 activation were promptly and equally inhibited [60] suggesting a proximal mechanism of INH-ODN action, possibly at the level of TLR9 receptor itself [61].

Further mapping studies in mouse B and non-B cells [54, 55] have established the following rules for TLR9-inhibition: (1) CpG motif, either methylated or unmethylated, is not required for inhibition; (2) three consecutive G nucleotides are necessary for inhibition; (3) 5′ end of an ODN is important for both stimulation and inhibition: TCC is optimal for stimulation, while CC(T) triplet is required for optimal inhibition; (4) the distance between the 5′ CC(T) and downstream GGG triplet should optimally be 3–5 nucleotides long; (5) the order of 5′CC(T)→GGG-3′ is critical as ODNs with the reverse order, for example, 5′GGG→CC(T)-3′, or with the reverse sequence are non-inhibitory; (6) the intervening sequence between the CC(T) and GGG elements contributes minimally to the overall ODN activity and can accept multiple modifications. (7) at the 3′ end of an INH-ODN, it is not the primary sequence but the length that contributes to the activity; (8) specific inhibition of TLR9-induced activation does not require intrachain and/or interchain Hoogsten hydrogen bonding between adjacent Gs, as deaza-substituted ODNs [59] and linear INH-ODNs incapable of making these bonds are equally effective TLR9 inhibitors [62].

ODNs containing the canonical mouse inhibitory motif for TLR9 (e.g., 2088, 2114, and 4024) were also active in human B cells, B cell lines, pDCs [63, 64], and in TLR9-transfected HEK cells [65]. However, extending INH-ODNs for 4-5 bases at the 5′ end significantly enhanced their activity for human cells. The activity in human cells does not depend on the ability of INH-ODN to either self-aggregate or directly bind to a stimulatory ODN. As in mouse TLR9-expressing cells, primary base sequence and backbone determine INH-ODN activity [65]. Even though TLR9 binds INH-ODNs as well as CpG-ODNs, the affinity for TLR9 does not correlate with the biologic activity (Ashman and Lenert, submitted for publication). Indeed, recent studies have shown that the sugar backbone 2-deoxyribose determines DNA recognition by TLR9, and base-free deoxyribose homopolymers may act as TLR9 agonists [66]. Phosphorothioate-modified deoxyribose has much higher affinity for both TLR7 and 9 compared to PO-deoxyribose, transforming these molecules into TLR7 and TLR9 antagonists [66]. Therefore, we hypothesize that some other molecule, not TLR9, must mediate sequence-specific recognition of INH-ODNs (unpublished data and [65]).

## 4. Concept of Class R and Class B INH-ODNs: Less Is Sometimes More

For our mapping studies, we initially used INH-ODN-2114 with the following sequence: 5′TCCTGGAGGGGAAGT-3′ ([59]; Table 1). This ODN is a very potent TLR9-antagonist in both human and mouse settings in vitro [54, 59, 65] as well as in the MRL-Fas *^lpr/lpr^* strain of lupus in vivo [67]. The PO variant of this ODN is active against PO-CpG-ODNs and bacterial DNA in B cells and mouse macrophages [68]. As PS ODNs bind to TLR9 with much higher affinity than PO-ODNs [69, 70], not surprisingly, PO-INH-ODN 2114 is at least 100-fold less potent against PS-CpG-ligands. Interestingly, a genomics search has shown that the optimal inhibitory sequence is severalfold more prevalent in mammalian DNA compared to bacterial *E.coli* DNA, suggesting a physiologic relevance of these findings [68]. Our INH-ODNs and similar ODNs developed by Stunz et al., Barrat et al., and Peter et al. have no inhibitory activity on TLRs 2, 3, 4, 5, and BCR-induced activation when used at concentrations up to 1 micromol [59, 71, 72]. The effects on TL7/8 will be discussed below. 

**Figure 1:**
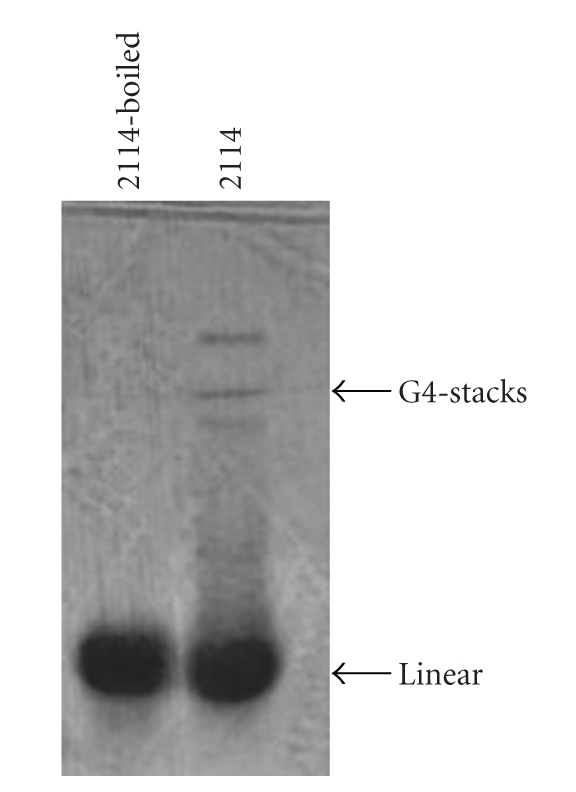
Class B INH-ODN 2114 can undergo G4-stacking in the presence of potassium ions. INH-ODN 2114 (1 *μ*g) was dissolved in a buffer containing potassium ions at 65 degrees for 10 minutes and then slowly cooled down to room temperature for 2 hours. For comparison, the same ODN was dissolved in Tris-EDTA, boiled for 10 minutes and rapidly cooled on ice. Electrophoretic mobility on 20% native PAGE gel is shown. Gel was stained with Stains All overnight.

Since INH-ODN-2114 can make (some) G4-stacks as poly-G ODNs [73] (Figure 1), the interpretation of these results may be complicated by nonspecific effects of G4 stacks on immune activation. As a matter of fact, TLR9-independent effects of ODN-2114 were seen in the model of intracellular *S. typhimurium* infection in TLR9-deficient bone marrow derived macrophages [74]. Therefore, in order to avoid any contribution from G4 aggregates to TLR9 inhibition, we created INH-ODN 4024 (TCCTGGATGGGAAGT) [63]. This ODN contains both CC(T) and GGG triplets and is as potent as INH-ODNs 2088 and 2114 for CpG-ODN-stimulated mouse B cells and macrophages [55, 63]. Further truncation of ODN-4024 resulted in the shortest active 12-mer INH-ODN 4084-F with the sequence 5′CCTGGATGGGAA3′ [62]. 

In order to better understand the role of secondary structures, for example, ability to make DNA duplexes or hairpins, we used INH-ODN 4084F as a template. We created 24 mer-ODNs in which the 4084F sequence was either at the 5′ or the 3′ end of the molecule, and was followed (or preceded) by 12 nucleotides complementary to the 4084F, making a complete palindrome (Table 1, INH-1, INH-4) [62]. We named these new TLR9-antagonists: Class R INH-ODNs (where “R” stands for restricted activity, [75]) as they showed similar inhibitory potency for TLR9-activated IFN-*α* producing dendritic cells (and macrophages/macrophage cell lines) as their linear analogues (Class B, broadly-active, Table 1, INH-18, INH-13), but were between 10–30-fold less active in resting mouse splenic (follicular) B cells irrespective of the outcome tested. These ODNs were also less potent in human peripheral blood B cells and in B cell lines [62]. Interestingly, even bigger potency difference (~100 fold) was observed when these ODNs were made with the natural phosphodiester backbone [62]. Similar to complete palindromes, ODNs having short 5′ or 3′ overhangs (up to 6 nucleotides long) were less active in B cells when compared to their linear analogues [62]. The difference in activity between Class R and B INH-ODNs in B cells could not be explained by differences in the uptake, but could depend on the ability of these ODNs to reach different TLR9-expressing compartments, for example, early versus late endosomes [62, 76–78]. We hypothesized, that in B cells, Class R INH-ODNs, similar to mammalian DNA, may have restricted access to late endolysosomes. Interestingly, similar to differences between Class R and B INH-ODNs, human naïve B cells and resting mouse follicular B cells poorly respond to complex TLR9 agonists, for example, double-stranded bacterial DNA and type A(D) CpG-ODNs which have a palindromic center and G-rich tails [20, 79–81]. Since the signal through the B cell receptor for antigen allows B cells to respond to a wider range of TLR9 ligands including complex TLR9-agonists [78, 82–86], we wondered whether the same principal holds true for Class R TLR9-antagonists. We studied this hypothesis in both autoimmune and nonautoimmune settings. We used autoreactive rheumatoid factor-specific AM14 B cells as a model for BCR/TLR9 cross talk [87]. AM14 B cells proliferate upon recognition of DNA/or RNA containing-immune complexes by their B cell receptor for antigen only if co-stimulated through the TLR7 or TLR9 [88, 89]. When AM14 B cells were stimulated with linear CpG-ODN ligands (e.g., with CpG-ODN 1826), similar to non-autoreactive B cells, Class R INH-ODNs were at least 10-fold less potent inhibitors compared to Class B INH-ODNs [62]. However, when DNA-containing immune complexes were used for stimulation, the potency of Class R INH-ODN increased for at least 10-fold equalizing that of Class B INH-ODNs [62]. Since in AM14 B cells, INH-ODNs fail to inhibit signaling through the BCR, or by LPS, we concluded that the increased potency of Class R INH-ODNs for BCR/TLR9 coactivated autoreactive B cells could be advantageous for selective targeting of autoimmune B cells in lupus. Indeed, contrary to our expectations, our in vivo studies in the MRL-Fas *^lpr/lpr^* strain showed that potent linear TLR9-specific antagonist (Class B INH-18) was surprisingly ineffective while treatment with palindromic Class R INH-1 resulted in improved survival and less renal pathology [62]. Furthermore, levels of anti-dsDNA and anti-Sm/RNP antibodies were significantly reduced and abnormal lymphoproliferation was halted. These results could be explained by the fact that TLR9 may have some protective, rather than pathogenic, effects in the MRL-Fas *^lpr/lpr^* strain of lupus mice. TLR9 may be critical for the induction of regulatory T cells in this strain as hypothesized by Wu and Peng [29]. Moreover, the principal cytokine involved in the pathogenesis of lupus in this strain appears to be IFN-*γ*, not IFN-*α*, as MRL-Fas *^lpr/lpr^* mice deficient in IFN-*α* receptor have more severe disease [90–92]. Other explanations are also possible, including selective effects of palindromic INH-ODNs on TLR7 activation, as TLR7 plays a well-proven role in the pathogenesis of MRL-Fas *^lpr/lpr^* lupus [28].

## 5. Telomeric TTAGGG Repeats as Immune Modulating Agents

Oligonucleotides containing repetitive TTAGGG motifs were developed by Klinman's group and were shown to have multiple effects on immune activation [61]. TTAGGG repeats are found in telomeric ends and physiologically protect mammalian chromosomes from degradation [93]. It appears that when our own DNA is released from cells, these telomeric regions are responsible for inhibitory effects of mammalian DNA [53, 61]. Indeed, DNA from telomerase-deficient mice is much less suppressive than the control DNA (reviewed in [94]). Synthetic ODNs containing TTAGGG repeats were capable of blocking the production of proinflammatory and TH1 cytokines induced not only with TLR9 ligands, but also with a variety of polyclonal activators and antigens [61, 94, 95]. For example, they were active against double-stranded RNA, peptidoglycan, and even against lipopolysaccharide (LPS) when IFN-*γ* production was measured as an outcome (reviewed in [94]). Interestingly, others have shown that similar G-rich ODNs can bind IFN-*γ* directly and act as aptamers [96]. In vivo, these ODNs showed a remarkable potential to prevent pathology in animal models of inflammatory arthritis induced by intra-articular injection of CpG-ODNs [97], spontaneous SLE in NZB/W mice [98], experimental uveitis [94], acute silicosis [99], and LPS-induced toxic shock [100]. Interestingly, while TTAGGG-ODNs were capable of preventing the development of nephritis in NZB/W mice, treatment of animals with established lupus nephritis did not stop the progression of the disease [98]. Authors concluded that these ODNs may be promising agents for treatment of a variety of autoimmune and inflammatory diseases, particularly when administered early in the course of the disease [94]. 

While the mechanism of action of these ODNs is incompletely understood, immunosuppressive ability of these ODNS was found to be heavily dependent on their ability to make complex structures, for example, G4-stacks. TTAGGG motifs may act, at least in part, by selectively binding to STAT1 and STAT4 and by blocking their subsequent phosphorylation [95, 100]. Interestingly, we were not able to observe any (inhibitory) effects of linear (non-G4-stack forming) Class B INH-ODNs on STAT signaling suggesting that different classes of INH-ODNs may act through different signaling pathways (data not shown). Others have shown that G-rich ODNs, similar to TTAGGG repeats, can also target another member of the STAT family, a STAT3 oncogene, with an IC(50) of 7 micromol [50, 51]. Other cellular targets of G-rich ODNs have also been identified, including scavenger receptors [73], nucleolin [101], and interestingly, a lupus autoantigen-Ku [102]. 

## 6. Combined TLR7/TLR9 Antagonists

Barrat-Coffman's group at Dynavax used our INH-ODN 2114 [54, 59] as a template for creating novel TLR9 inhibitors, such as IRS 869 (TCCTGGAGGGGTTGT). They studied their effects in human and mouse B cells and in IFN-*α*-producing plasmacytoid dendritic cells [64]. Noticeably, the ODN variant they used (IRS 869) differed from INH-ODN 2114 only by two A → T substitutions at the 3′ end where the number of nucleotides, but not the primary sequence, matters [54, 55]. They found that 4 contiguous G residues were essential for TLR9 inhibition [64]. Since IRS 869 could make G4-stacks, they also studied the contribution of primary sequence versus G4-aggregates to the inhibitory activity. Similar to our results, they observed that linear sequence, not the G-aggregate, was responsible for TLR9 inhibition in human B cells [64]. They further showed that these ODNs were efficacious in vivo in a model of d-galactosamine + CpG-ODN-induced sepsis. INH-ODNs prevented massive systemic inflammation and cytokine release responsible for sepsis in this model [64]. This effect was confirmed in a recent study by Plitas et al. in a model of polymicrobial sepsis [103].

Barrat's group subsequently developed short INH-ODNs that preferentially block TLR7-induced innate activation. The prototypic TLR7 antagonist IRS 661 contained 5 GC motifs equally spaced within the complete palindrome [104]. This ODN specifically blocked small TLR7/8 agonist (R848)-induced splenocyte IL-6 secretion, but was ineffective against TLR9-ligand induced activation. In their hands, TLR9-specific antagonists (e.g., IRS 869) failed to block TLR7 (R848)-dependent activation. The same group also developed IRS 954 (TGCTCCTGGAGGGGTTGT) which was capable of simultaneously blocking both TLR7- and TLR9-dependent activations. Combined TLR7 and TLR9 inhibitors suppressed IFN-*α* induction by either ultraviolet-light irradiated HSV (DNA), inactivated influenza virus (ss RNA virus), or by RNA-containing immune-complexes. IRS 954 also slowed down the progression of spontaneous lupus in the NZB/W-F1 strain of lupus mice and reduced the production of multiple autoantibodies (e.g., anti-dsDNA, antinucleosome, anti-Sm, and anti-RNP antibodies) [104]. Interestingly, the control ODN used in their study lacked the TLR9 motif but contained the TGC motif which was buried in the interior of the molecule. Since this control ODN was apparently ineffective, one may wonder which pathway (TLR7 or 9?) was a primary target of INH-ODNs in this model [104]. Interestingly, NZB/W-F1 mice, similar to SLE patients, constitutively express high levels of IFN-*α* regulated genes. Moreover, treatment with IFN-*α* accelerates disease, while mice deficient in the IFN-*α* receptor develop less severe disease with delayed onset [105, 106]. 

In contrast to the results from Barrat's group, several groups, including our own, have shown that PS ODNs including INH-ODNs (but not PO INH-ODNs) have backbone-dependent and sequence-independent effects on TLR7 activation induced by either RNA-containing immune complexes, or by small TLR7 agonists like R837 and CL075 [57, 62, 89, 107–109]. However, in contrast to RNA-immune complex-induced activation of pDCs, R848-induced B cell activation is relatively difficult to inhibit with TLR9-specific antagonists, but remains sensitive to TGC-containing ODNs, clarifying this controversy (Lenert, unpublished data). 

## 7. Proposed Mechanisms of INH-ODN Action

Diversity of published sequences for TLR9 inhibition suggests a possibility of different sites and mechanisms responsible for their inhibitory action. For example, INH-ODNs may act as nonsequence specific competitors for receptor-mediated endocytosis or phagocytosis. This effect may depend on a cell type, presence of scavenger receptors (e.g., CXCL16, SR-A, CD36, MARCO) and on the overall length of INH-ODNs, as well as on their ability to make G4 stacks [73, 110, 111]. In general, longer and G-rich ODNs are better taken up by macrophages than shorter ODNs. The opposite is true in B cells [111]. A second possibility is inhibition of TLR9-trafficking or TLR9-processing into functionally active product [38]. A third mechanism may involve competitive antagonism at the level of TLR9-expressing endosome. For example, INH-ODNs may bind TLR9 and prevent it from undergoing a conformational change critical for recruiting MyD88 [40]. Further mechanism may include inhibition of endolysosomal acidification (similar to chloroquine action) or pharmacologic inhibition of various proteases, for example, cathepsins [112] or asparaginyl endopeptidases. Recently discovered cysteine protease asparaginyl endopeptidase is important for TLR9 processing in DCs, but not in macrophages [39]. There is also a possibility that certain INH-ODNs may work downstream of the TLR9 (and TLR7) for example, at the level of STATs 1, 3, and 4. Finally, while TLR9-specific inhibitors may block TLR7-induced activation via their backbone sugars [66], TLR9 itself is not needed for this inhibition (Ashman et al., unpublished observations). 

## 8. Revised Classification of INH-ODNs

A few years ago we proposed a classification of INH-ODNs into two major categories: Class B and Class R [75]. Class B INH-ODNs are broadly reactive linear ODNs that potently block CpG-induced activation in all TLR9-expressing cells. On the other hand, Class R INH-ODNs are capable of making significant secondary structures and are less active in resting B cells. We initially classified all complex INH-ODNs into the Class R category [75]. However, it is now clear that a substantial difference exists between telomeric and palindromic ODNs in terms of their ability to make G4-stacks and their TLR9-specificity. Therefore, in this revised classification we define a new category of INH-ODNs—Class G. Class G INH-ODNs contain multiple G3 triplets (like telomeric repeats) or G4 tetrads and are capable of making large G-aggregates. They inhibit not only signaling through the TLR9, but also activation through other TLRs. They are directly proapoptotic in tumor cells and can additionally block stimulation of other immune cells, for example, T cells, nonspecifically. Table 2 depicts the most important characteristics of these three categories of TLR9 antagonists.

## 9. Conclusions

At least three different classes of INH-ODNs have recently been developed. While all these ODNs can block TLR9-dependent activation, and exhibit backbone-dependent effects on TLR7 stimulation, depending on their size and ability to make G4-stacks, they may have additional cellular targets. For example, telomeric TTAGGG repeats and poly-G ODNs can be classified as Class G INH-ODNs. Compared to other classes they are relatively TLR9-nonspecific. They can block phosphorylation and nuclear translocation of multiple members of the STAT family, for example, STAT 1, 3, and 4. They can additionally interact with scavenger receptors on macrophages, Ku-autoantigen, and with nucleolin. They showed potent immune-modulatory effects in animal models of lupus in the NZB/W-F1 strain [98], and in various experimental models of arthritis, sepsis, uveitis, and silicosis (reviewed in [94]). Because of their cellular and target promiscuity, they can be more immmunosuppressive than other classes of INH-ODNs. Thus, chronic treatment with Class G INH-ODNs may potentially lead to enhanced susceptibility to infection, even though the phenotype of mutated mice including those lacking the functional transporter molecule UNC93B1 is relatively mild [37]. Class B INH-ODNs are strictly linear ODNs unable to make significant secondary structures. They require a 5′CC(T)→GGG-3′ motif to block TLR9-induced activation in all responding cells, both in humans and in mice. Interestingly, they are less protective in the MRL-Fas *^lpr/lpr^* strain when compared to Class R INH-ODNs. They may find applications for prevention/treatment of TLR9-dependent microbial sepsis and chronic inflammation. When the number of consecutive Gs in a linear INH-ODN is increased from 3 to 4-5, this increases a chance for G4 stacking and for nonspecific effects on immune activation. Finally, Class R INH-ODNs are longer (20–28 mer) ODNs capable of either dimerizing or making hairpins. This property of Class R INH-ODNs depends on ODN-concentration, presence of ions, and on temperature. They are very potent suppressors of TLR9-induced activation in pDCs and macrophages, but are 10–30-fold less potent in human naïve B cells and mouse follicular B cells [62]. This cell selectivity of palindromic INH-ODNs is independent of the G4-stacking. BCR cross-linking increases their potency for TLR9-activated B cells for at least 10-fold making them ideal candidates for targeting dsDNA-, nucleosome-, or RF-specific autoreactive B cells. 

All three classes of TLR9-antagonists have sequence-independent backbone-dependent effects on TLR7 (and possibly TLR3?) stimulation. TGC triplets may additionally increase the potency of an INH-ODN for the TLR7 pathway [71]. Literature search shows that classes B and G INH-ODNs and combined TLR7/9 inhibitors are effective in animal models of lupus [62, 67, 98, 104, 113]. We envision their future use as therapeutic agents for human lupus. 

## Figures and Tables

**Table 1:** Oligonucleotides used in this study.

No.	Sequence	Class	Reference
2088	TCCTGGCGGGGAAGT	B/G	[58, 59]
2114	TCCTGGAGGGGAAGT	B/G	[58, 59]
4024	TCCTGGATGGGAAGT	B	[54, 55]
4084F	CCTGGATGGGAA	B	[62]
INH-1	CCTGGATGGGAATTCCCATCCAGG	R	[62]
INH-4	TTCCCATCCAGGCCTGGATGGGAA	R	[62]
INH-13	CTTACCGCTGCACCTGGATGGGAA	B	[62]
INH-18	CCTGGATGGGAACTTACCGCTGCA	B	[62]
Poly-G	GGGGGGGGGGGGGGGGGGGG	G	[47]
A151 (telomeric)	TTAGGGTTAGGGTTAGGGTTAGGG	G	[61]
GpG	TGACTGTGAAGGTTAGAGATGA	B	[56]
G-ODN	CTCCTATTGGGGGTTTCCTAT	B/G	[72]
IRS-869	TCCTGGAGGGGTTGT	B/G	[64]
IRS-661	TGCTTGCAAGCTTGCAAGCA	R/TLR7 specific	[71]
IRS-954	TGCTCCTGGAGGGGTTGT	B/TLR7/9 specific	[71]

**Table 2:** Classification of inhibitory oligonucleotides.

Class	Characteristics	Prototype	TLR9 inhibition in B cells	TLR9 inhibition in DC/MΦ	TLR7 inhibition (backbone effect)	Inhibition of other signaling pathways	Reference
G	G4-stacking	TTAGGGn	+	+++	++	+++	[61]
R	Palindromic, Short 5′ or 3′overhangs	INH-1	+	+++	++	—	[62]
B	Linear	INH-18	+++	+++	++	—	[62]

## Abbreviations

TLR: Toll-like receptor
INH-ODN: Inhibitory oligonucleotide
BCR: B cell receptor for antigen
SLE: Systemic lupus erythematosus.
